# Association of tirzepatide and the risk of suicide in a real-world cohort

**DOI:** 10.3389/fpsyt.2025.1626103

**Published:** 2025-11-17

**Authors:** Wei-Shin Yu, Jing-Yang Huang, Shih-Chang Lo, Chien-Ning Huang, Yi-Sun Yang, Edy Kornelius

**Affiliations:** 1 Chung Shan Medical University Hospital, Department of Internal Medicine, Division of Endocrinology and Metabolism, Taichung, Taiwan; 2 Chung Shan Medical University Hospital, Department of Medical Research, Taichung, Taiwan; 3 Chung Shan Medical University, School of Medicine, Taichung, Taiwan; 4 Chung Shan Medical University, Institute of Medicine, Taichung, Taiwan

**Keywords:** tirzepatide, suicide, obesity, psychiatry, diabets mellitus

## Abstract

**Background:**

Glucagon-like peptide-1 (GLP-1) receptor agonists, including tirzepatide, are increasingly prescribed for obesity and type 2 diabetes mellitus. Amid safety concerns raised by regulatory agencies regarding potential associations with suicidal ideation or behavior, real-world data on psychiatric risks are critically needed. This study aims to evaluate the association between tirzepatide and suicidal ideation or attempts compared to other anti-obesity medications in a real-world population.

**Methods:**

A retrospective cohort study using data from the TriNetX US Collaborative Network, covering 66 healthcare organizations from May 2022 to April 2024. A total of 226,060 patients with overweight or obesity, including 29% with type 2 diabetes, were included. Propensity score matching was applied to compare tirzepatide (N=42,600) with non-GLP-1 anti-obesity medications (N=183,460).

**Results:**

After matching, 16,321 pairs were analyzed. The mean age was 48.4 years, and 69.1% were female. Over a median follow-up of 365 days, 17 tirzepatide patients and 33 control patients experienced suicidal ideation or attempts. Tirzepatide was associated with a 48% lower risk of suicide ideation or attempts (aHR: 0.52, 95% CI: 0.28–0.91; P=0.001). Subgroup analyses by BMI, HbA1c, and GFR indicated consistent trends of lower risk. While these findings suggest a potential protective association, the observational nature of this study precludes definitive conclusions regarding causality.

**Conclusions:**

Tirzepatide use was associated with a lower observed risk of suicidal ideation or suicide attempts compared with other anti-obesity medications. Long-term studies are needed to confirm these findings.

## 1 Introduction

Obesity is a prevalent and complex condition that significantly contributes to morbidity and mortality worldwide (1), with associated comorbidities including type 2 diabetes (T2D), cardiovascular disease, and a range of psychological disorders such as depression and anxiety (2–6). Despite advances in lifestyle and pharmacological interventions, effective long-term management of obesity remains challenging. Recently, tirzepatide, a novel dual glucose-dependent insulinotropic polypeptide (GIP) and glucagon-like peptide-1 (GLP-1) receptor agonist, has demonstrated considerable efficacy in improving glycemic control and promoting weight reduction in patients with obesity (7–9). Its dual action mechanism offers a promising approach for the treatment of obesity and related metabolic conditions.

However, the psychological impact of tirzepatide, particularly its potential association with mental health outcomes, remains underexplored. This is of particular concern given the European Medicines Agency’s (EMA) recent alert regarding a possible link between GLP-1 receptor agonists and an increased risk of suicidal ideation and behavior (10, 11). Although randomized controlled trials (RCTs) have not definitively established a psychiatric risk profile for other GLP-1 receptor agonists such as liraglutide and semaglutide, observational studies have largely indicated no increased risk of suicide with their use (12–15). Nevertheless, only a few studies to date have specifically evaluated the risk of suicidal ideation or attempts associated with tirzepatide, leaving a significant gap in the literature that warrants further investigation (11). Additionally, it remains uncertain whether weight loss alone, regardless of the specific intervention, exerts a protective or detrimental effect on suicide risk, as prior data on this topic are conflicting (6, 16, 17).

In light of these concerns, this study aims to investigate the potential association between tirzepatide use and the risk of suicide in patients with obesity. By addressing this gap, we seek to contribute to a more comprehensive understanding of the safety profile of tirzepatide and inform clinical decision-making.

## 2 Methods

### 2.1 Data source

This study utilized data from the TriNetX US Collaborative Network, a comprehensive real-world data platform that aggregates information from 66 healthcare organizations, including academic medical centers, community hospitals, and large integrated healthcare systems. The TriNetX platform collects clinical data directly from electronic health records (EHRs), billing systems, and disease registries, encompassing a wide range of demographics, diagnoses, treatments, and outcomes.

The credibility of the TriNetX platform is supported by its extensive use in peer-reviewed research and clinical studies, offering real-time access to a broad patient population across diverse geographic and healthcare settings (18, 19). The accuracy and completeness of its datasets are maintained through regular data quality assessments, leveraging the platform’s automated data cleaning algorithms and collaboration with healthcare institutions to ensure the reliability of the clinical information obtained.

### 2.2 Study population

The study population consisted of patients with overweight or obesity who were prescribed either tirzepatide or other anti-obesity medications, including bupropion, naltrexone, orlistat, topiramate, phentermine, or setmelanotide. Patients prescribed tirzepatide were designated as cases, while those receiving other anti-obesity medications served as active controls. This active comparator design was selected to ensure that all patients had similar motivations for weight management and shared comparable baseline characteristics, thus allowing for a more accurate comparison of outcomes.

The index date for the case group was defined as the date of the first tirzepatide prescription, while for the control group, it was the date of the first prescription for one of the aforementioned anti-obesity medications. Patients with a documented history of suicidal ideation or suicide attempts prior to the index date were excluded to minimize potential confounding, because the presence of prior suicidality can indicate a distinct clinical pathway and risk profile that might obscure new-onset events. Additionally, patients with prior prescriptions of other GLP-1 receptor agonist medications were excluded to eliminate potential confounding effects.

The study period began in May 2022 and concluded in April 2024, with a maximum follow-up period of one year per patient to ensure consistent comparisons between the tirzepatide and control groups. The median follow-up time was 365 days. We recognize that one year may be a relatively short period for assessing long-term psychiatric outcomes; however, this timeframe was selected to maintain uniform data availability in TriNetX and to reduce time-varying confounders.

### 2.3 Study outcome

The primary endpoint of this study was the first occurrence of a composite outcome, including suicidal ideation or suicide attempts, after the index date. These diagnoses were obtained from clinical visits documented within the EHRs, ensuring a high degree of reliability, as they were made by qualified healthcare professionals during clinical assessments.

### 2.4 Study covariates

We carefully selected a comprehensive set of covariates for analysis, ensuring that these variables were obtained either on or within one year prior to the index date. The covariates included a range of demographic factors such as age, sex, ethnicity, race, and marital status. Socioeconomic status, family history of mental and behavioral disorders, and lifestyle-related factors were also considered to account for potential influences on suicide risk.

Baseline clinical characteristics were incorporated into the analysis, including body mass index (BMI), serum creatinine, and HbA1c levels. Pre-existing medical conditions associated with an increased risk of suicide, such as mood disorders, anxiety, schizophrenia, alcohol use disorder, and chronic pain, were thoroughly assessed. In addition, the analysis considered patients’ previous medical interventions, including a history of bariatric surgery and prescriptions for antidepressants, antidiabetic medications, and other relevant therapies. Detailed coding for this study is provided in **Supplementary Table 1**.

### 2.5 Statistical analysis

We applied 1:1 propensity score matching (PSM) using greedy nearest neighbor matching with a 0.1 caliper to balance baseline characteristics between the tirzepatide and control groups. This caliper width is the default PSM setting in TriNetX, and is commonly used to optimize balance while retaining a sufficiently large sample. However, we acknowledge that such a caliper may exclude certain patients, potentially introducing selection bias and reducing generalizability. Matching was performed for all covariates to minimize selection bias.

Kaplan-Meier analysis was used to estimate the probability of the primary outcome over time, with censoring applied for patients exiting the cohort. Hazard ratios (HRs) and 95% confidence intervals (CIs) were calculated to compare time-to-event rates between groups. Cox proportional hazards models were employed for this comparison. A p-value of<0.05 as statistically significant. All analyses were performed within the TriNetX platform using its default analytical software.

For subgroup analyses by sex, ethnicity, race, age, BMI, HbA1c, and eGFR, we did not apply formal multiple-comparison corrections due to the low event rate and the exploratory nature of these analyses. Additionally, we did not perform more advanced sensitivity analyses (e.g., Bayesian modeling or inverse probability weighting [IPW]) due to TriNetX constraints and the rarity of events, which may limit statistical power and generalizability. No imputation was performed for missing data. PSM was conducted using available values, and patients with missing covariates were excluded from that variable-specific analysis within the TriNetX platform.

## 3 Results

### 3.1 Baseline demographic

**Figure 1:**
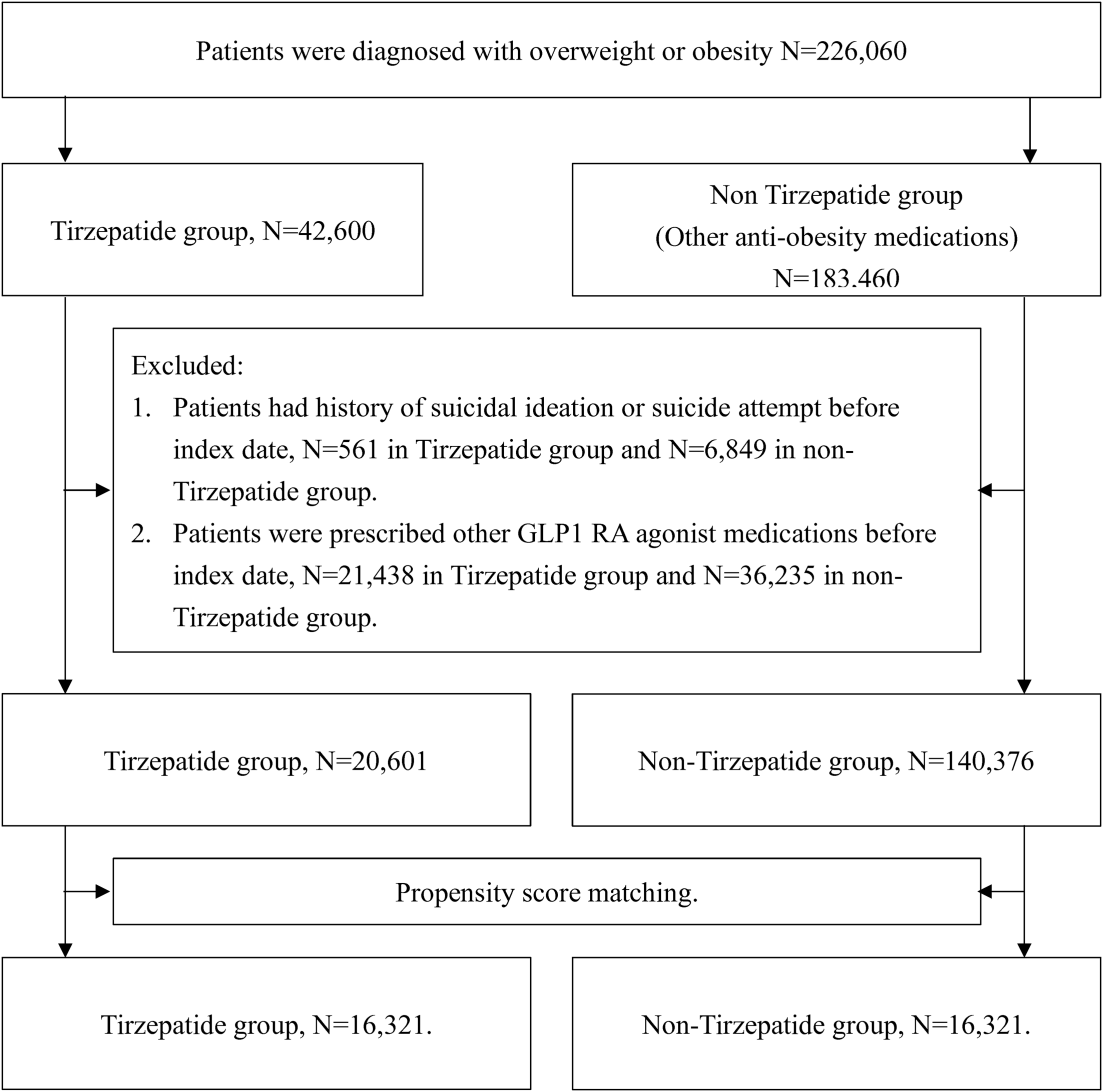
Flow diagram of this study.

A total of 226,060 patients with overweight or obesity were identified from the TriNetX database, comprising 42,600 patients prescribed tirzepatide and 183,460 patients prescribed non-tirzepatide anti-obesity medications. Following the application of exclusion criteria, including the removing patients with a history of suicidal ideation, suicide attempts, or prior use of any GLP-1 receptor agonists, 20,601 patients in the tirzepatide group and 140,376 patients in the control group were eligible for analysis (**Figure 1**).

Before PSM, significant differences were observed between the tirzepatide and control groups in terms of age, BMI, and comorbid conditions. To address these imbalances, 1:1 PSM was performed, resulting in 16,321 matched pairs based on all pre-specified covariates. After matching, baseline characteristics, including age, sex, ethnicity, race, BMI, HbA1c, and pre-existing mental health conditions, were well balanced between the two groups, with no statistically significant differences (standardized mean difference [SMD]< 0.1 for all comparisons; **Table 1**).

**Table 1:** Baseline characteristics before and after propensity score matching.

	Before PSM			After PSM		
	Tirzepatide	Non-tirzepatide	SMD	Tirzepatide	Non-tirzepatide	SMD
Total number	N = 20,601	N = 140,376		N = 16,321	N = 16,321	
Age at index, Mean ±SD	49.5 ± 13.0	47.1 ± 15.1	0.1677	48.4 ± 13.0	48.5 ± 14.5	0.0115
Sex (%)						
Female	13351(64.8%)	100369(71.5%)	0.1440	11249(68.9%)	11282(69.1%)	0.0044
Male	5794(28.1%)	30891(22.0%)	0.1415	3917(24.0%)	4056(24.9%)	0.0198
Ethnicity (%)						
Hispanic or Latino	1311(6.4%)	11067(7.9%)	0.0591	1054(6.5%)	1029(6.3%)	0.0063
Not Hispanic or Latino	13968(67.8%)	101409(72.2%)	0.0970	11231(68.8%)	11350(69.5%)	0.0158
Unknown	5322(25.8%)	27900(19.9%)	0.1423	4036(24.7%)	3942(24.2%)	0.0134
Race (%)						
Asian	318(1.5%)	1430(1.0%)	0.0467	230(1.4%)	146(0.9%)	0.0482
Black	2812(13.7%)	19155(13.6%)	0.0001	2144(13.1%)	2050(12.6%)	0.0172
White	14437(70.1%)	95616(68.1%)	0.0425	11506(70.5%)	11710(71.7%)	0.0276
Unknown	2305(11.2%)	17039(12.1%)	0.0296	1858(11.4%)	1755(10.8%)	0.0201
Marital status (%)						
Never married	3175(15.4%)	25824(18.4%)	0.0797	2583(15.8%)	2344(14.4%)	0.0409
Divorced	1179(5.7%)	8282(5.9%)	0.0076	970(5.9%)	775(4.7%)	0.0531
Widowed	615(3.0%)	4437(3.2%)	0.0102	491(3.0%)	435(2.7%)	0.0207
Adverse socioeconomic determinants of health (%)	392(1.9%)	4695(3.3%)	0.0903	338(2.1%)	342(2.1%)	0.0017
Personal history of psychological trauma (%)	10(0.0%)	182(0.1%)	0.0272	10(0.1%)	10(0.1%)	0.0000
Family history of mental and behavioral disorders (%)	30(0.1%)	541(0.4%)	0.0466	29(0.2%)	36(0.2%)	0.0096
Lifestyle-related problems (%)	595(2.9%)	7704(5.5%)	0.1301	511(3.1%)	509(3.1%)	0.0007
Pre-existing medical conditions (%)						
Depression	3355(16.3%)	43516(31.0%)	0.3516	3116(19.1%)	3277(20.1%)	0.0249
Mood disorders, including bipolar disorder	4712(22.9%)	59124(42.1%)	0.4199	4328(26.5%)	4516(27.7%)	0.0259
Anxiety, dissociative, somatoform and other nonpsychotic mental disorders, including posttraumatic stress disorder	5905(28.7%)	57942(41.3%)	0.2668	5267(32.3%)	5479(33.6%)	0.0276
Schizophrenia, schizotypal, delusional and other non-mood psychotic disorders	73(0.4%)	1470(1.0%)	0.0831	67(0.4%)	70(0.4%)	0.0028
Behavioral disorders, including sleep disorders	892(4.3%)	8378(6.0%)	0.0742	778(4.8%)	825(5.1%)	0.0133
Disorders of adult personality and behavior, including impulse and gender identity disorders	67(0.3%)	1251(0.9%)	0.0728	62(0.4%)	72(0.4%)	0.0096
Symptoms and signs involving an emotional state	176(0.9%)	2449(1.7%)	0.0787	161(1.0%)	168(1.0%)	0.0043
Sleeping disorders including insomnia	5592(27.1%)	38350(27.3%)	0.0039	4320(26.5%)	4283(26.2%)	0.0051
Chronic pain	2770(13.4%)	23453(16.7%)	0.0912	2319(14.2%)	2327(14.3%)	0.0014
Alcohol use disorder	206(1.0%)	4687(3.3%)	0.1611	191(1.2%)	236(1.4%)	0.0243
Tobacco use disorder	1156(5.6%)	16137(11.5%)	0.2116	1002(6.1%)	954(5.8%)	0.0124
Opioid use disorder	116(0.6%)	2120(1.5%)	0.0936	108(0.7%)	125(0.8%)	0.0124
Cannabis use disorder	72(0.3%)	1623(1.2%)	0.0934	65(0.4%)	67(0.4%)	0.0019
Cocaine use disorder	10(0.0%)	562(0.4%)	0.0744	10(0.1%)	14(0.1%)	0.0090
Other stimulant-related disorders	26(0.1%)	683(0.5%)	0.0652	23(0.1%)	24(0.1%)	0.0016
Other psychoactive substance-related disorders	60(0.3%)	1398(1.0%)	0.0882	56(0.3%)	59(0.4%)	0.0031
Type 2 diabetes mellitus	8960(43.5%)	18229(13.0%)	0.7203	4734(29.0%)	5036(30.9%)	0.0404
Cancer	2947(14.3%)	20451(14.6%)	0.0075	2310(14.2%)	2299(14.1%)	0.0019
Traumatic brain injury	109(0.5%)	1323(0.9%)	0.0484	95(0.6%)	98(0.6%)	0.0024
Previous medication prescription or procedures (%)						
Bariatric surgery	650(3.2%)	7255(5.2%)	0.1009	592(3.6%)	605(3.7%)	0.0042
Antidepressants	6592(32.0%)	93428(66.6%)	0.7366	6392(39.2%)	6827(41.8%)	0.0543
Antipsychotics	1334(6.5%)	18789(13.4%)	0.2326	1195(7.3%)	1247(7.6%)	0.0121
Antiepileptics	3264(15.8%)	56375(40.2%)	0.5626	3164(19.4%)	3327(20.4%)	0.0250
Benzodiazepine-derivative sedatives or hypnotics	3572(17.3%)	34063(24.3%)	0.1713	3046(18.7%)	3223(19.7%)	0.0275
Esketamine	0(0.0%)	18(0.0%)	0.0160	0(0.0%)	0(0.0%)	0.0000
Ketamine	319(1.5%)	3901(2.8%)	0.0846	269(1.6%)	283(1.7%)	0.0067
Lithium	10(0.0%)	71(0.1%)	0.0009	10(0.1%)	10(0.1%)	0.0000
Bupropion	1824(8.9%)	70551(50.3%)	1.0182	1785(10.9%)	5689(34.9%)	0.5939
Naltrexone	267(1.3%)	11238(8.0%)	0.3227	261(1.6%)	1055(6.5%)	0.2492
Phentermine	1189(5.8%)	43173(30.8%)	0.6833	1128(6.9%)	8341(51.1%)	1.1150
Orlistat	13(0.1%)	621(0.4%)	0.0756	12(0.1%)	143(0.9%)	0.1170
Topiramate	808(3.9%)	37309(26.6%)	0.6640	798(4.9%)	2492(15.3%)	0.3500
Insulin	1996(9.7%)	9785(7.0%)	0.0985	1352(8.3%)	1348(8.3%)	0.0009
Metformin	5187(25.2%)	13624(9.7%)	0.4165	3044(18.7%)	3262(20.0%)	0.0338
Alpha glucosidase inhibitors	19(0.1%)	51(0.0%)	0.0221	13(0.1%)	14(0.1%)	0.0021
DPP-4 inhibitors	510(2.5%)	998(0.7%)	0.1413	264(1.6%)	300(1.8%)	0.0169
SGLT2 inhibitors	1306(6.3%)	2057(1.5%)	0.2537	614(3.8%)	722(4.4%)	0.0334
Sulfonylureas	915(4.4%)	1748(1.2%)	0.1932	477(2.9%)	520(3.2%)	0.0153
Thiazolidinediones	210(1.0%)	398(0.3%)	0.0916	108(0.7%)	128(0.8%)	0.0145
BMI, kg/m^2^ (%)						
<40.0	9689(47.0%)	84971(60.5%)	0.2733	8052(49.3%)	8067(49.4%)	0.0018
≥40.0	7324(35.6%)	39398(28.1%)	0.1612	5575(34.2%)	5556(34.0%)	0.0025
Creatinine, mg/dL (%)						
<1.5	14580(70.8%)	92482(65.9%)	0.1053	11255(69.0%)	11389(69.8%)	0.0178
1.5-2.0	577(2.8%)	4375(3.1%)	0.0186	433(2.7%)	421(2.6%)	0.0046
≥2.0	537(2.6%)	4707(3.4%)	0.0439	418(2.6%)	406(2.5%)	0.0047
HbA1c, % (%)						
<7.0	9964(48.4%)	55849(39.8%)	0.1735	8037(49.2%)	8058(49.4%)	0.0026
7.0-8.9	3135(15.2%)	4285(3.1%)	0.4320	1403(8.6%)	1621(9.9%)	0.0461
≥9.0	1702(8.3%)	2224(1.6%)	0.3124	691(4.2%)	818(5.0%)	0.0371
eGFR, CKD-EPI (%)						
<30.0	479(2.3%)	4385(3.1%)	0.0491	382(2.3%)	369(2.3%)	0.0053
30.0-59.9	1324(6.4%)	11335(8.1%)	0.0636	1027(6.3%)	1034(6.3%)	0.0018
≥60.0	10455(50.8%)	69406(49.4%)	0.0261	8101(49.6%)	8196(50.2%)	0.0116

PSM, propensity score matching; SMD, standardized mean difference; BMI, body mass index; eGFR, estimated glomerular filtration rate, The Chronic Kidney Disease Epidemiology Collaboration; DPP-4, Dipeptidyl peptidase 4; SGLT2, Sodium-glucose cotransporter-2.

The mean age of the study population was 48.4 years, with females comprising 69.1% of the cohort. The racial distribution was well-balanced between groups, with 71% identifying as White, 13% as Black or African American, and 1.4% as Asian. Regarding lifestyle-related factors, 3.1% of patients in both groups had documented issues such as smoking (6%) or alcohol use (1.2%). Pre-existing mental health conditions were prevalent, with 19.1% diagnosed with depression, 26.5% with mood disorders, and 32.3% with anxiety disorders. In terms of metabolic health, 49.2% of patients had baseline HbA1c levels<7%, and 50% had an estimated glomerular filtration rate (eGFR) >60 mL/min/1.73 m². Detailed baseline characteristics before and after matching are presented in **Table 1**.

### 3.2 Primary outcome

**Figure 2:**
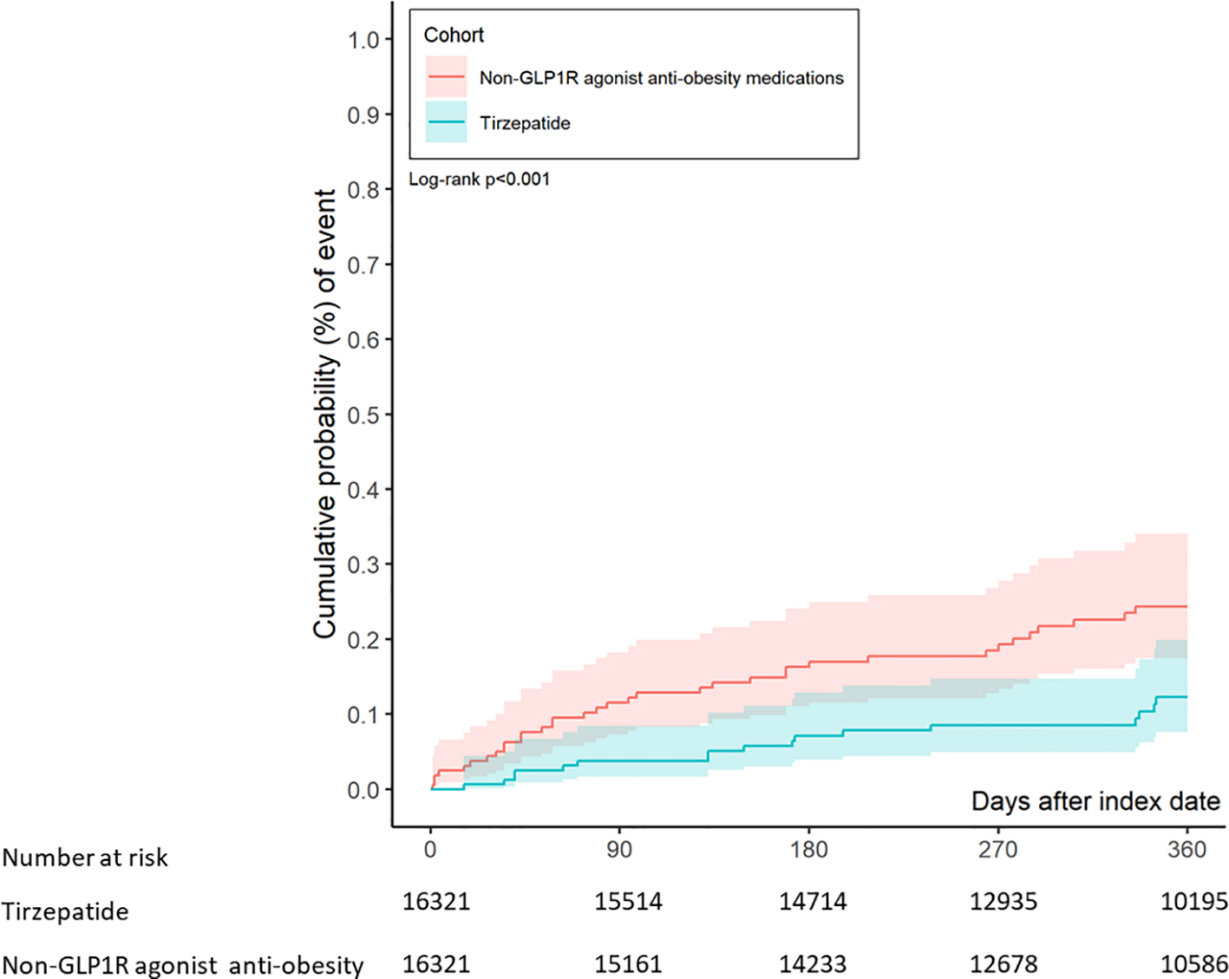
Kaplan-Meier chart evaluating the risk of suicidal ideation or suicide attempts in patients treated with tirzepatide and other anti-obesity medications.

During a median follow-up of 365 days, the composite primary endpoint of suicidal ideation or suicide attempts occurred in 17 patients in the tirzepatide group and 33 patients in the control group. This corresponds to an absolute rate of 1.04 cases per 1000 individuals in the tirzepatide group and 2.02 per 1000 in the control group, yielding an absolute risk reduction (ARR) of 0.98 per 1000 (NNT=1020). The adjusted hazard ratio (aHR) for the tirzepatide group compared to the control group was 0.52 (95% CI: 0.28–0.91, p = 0.001), indicating a significantly decreased risk of suicidal ideation or attempts in patients treated with tirzepatide. **Figure 2** shows that Kaplan-Meier analysis demonstrated a significant reduction in the risk of suicidal ideation or attempts in the tirzepatide group compared to the control group (log-rank p< 0.001).

Further analysis of suicidal ideation or attempts at 3, 6, and 12 months revealed adjusted hazard ratios of 0.04 (95% CI: 0.02–0.08), 0.07 (95% CI: 0.04–0.13), and 0.12 (95% CI: 0.08–0.20), respectively, demonstrating a consistent reduction in risk over time in the tirzepatide group compared to the control group (**Table 2**).

**Table 2:** The risk of suicidal ideation or suicide attempt in patients with tirzepatide.

	Tirzepatide	Non-tirzepatide	*P* value
Patients in Cohort, N	16,321	16,321	
Median follow-up, Days (IQR)	365 (77)	365 (81)	
Events within 1-year, n	17	33	
Incidence Probability, % (95% CI)			
at 3 months	0.04(0.02-0.08)	0.12(0.07-0.18)	
at 6 months	0.07(0.04-0.13)	0.17(0.12-0.25)	
at 1 year	0.12(0.08-0.20)	0.24(0.17-0.34)	
aHR (95% CI)	0.51 (0.28-0.91)	Reference	<0.001

aHR, adjusted hazard ratio.

**Figure 3:**
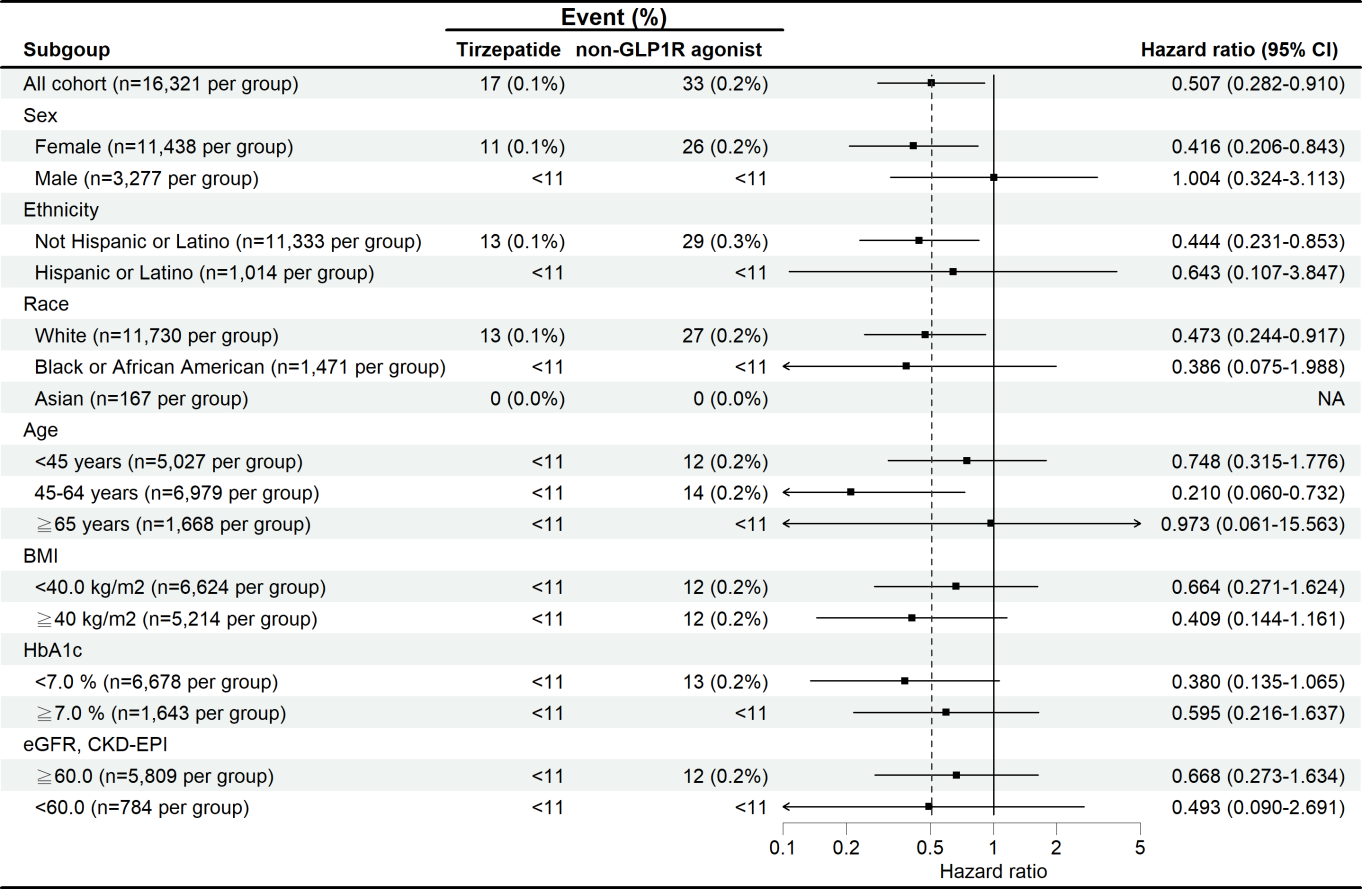
Subgroup analysis of the risk of suicidal ideation and suicide attempts in patients treated with tirzepatide compared with non-GLP-1 receptor agonist anti-obesity medications.

For subgroup analysis, the number of events was too small to conduct robust analyses, with most subgroups having fewer than 10 events, as shown in **Figure 3**. Stratified analyses by sex, ethnicity, race, age, BMI, HbA1c, and eGFR yielded consistent findings across all subgroups. Notably, female patients and those aged 45–64 years demonstrated directionally lower risks compared with their counterparts, although these findings did not reach statistical significance. In accordance with privacy protection protocols, when the number of outcomes in any subgroup was less than 10, the result was rounded up to 10 (**Figure 3**). Because of the limited statistical power, these subgroup results should be interpreted with caution.

## 4 Discussion

This study provides the first evidence supporting the safety of tirzepatide in relation to suicidal ideation and attempts. Over a median follow-up of 365 days, tirzepatide was associated with a 48% lower risk of suicidal ideation or attempts (aHR: 0.52, 95% CI: 0.28–0.91, p = 0.001). The consistent risk reduction observed at 3, 6, and 12 months further indicates a potential protective effect of tirzepatide over time.

Although the primary focus of our study was on patients with overweight or obesity, approximately 29% of the cohort also had T2D, a significant subgroup due to the well-established link between T2D, psychiatric disorders, and obesity. Our study demonstrated a consistently lower risk of suicide attempts in these patients. Subgroup analyses exploring the association between BMI, HbA1c, and GFR indicated that lower suicide ideation and attempt risks were observed across these metabolic variables. Despite the overall low incidence of suicide-related events, patients with better-controlled HbA1c levels and higher GFR appeared to have a reduced risk, highlighting the potential mental health benefits of improved glycemic control and kidney function in this population. Additionally, subgroup analyses (**Figure 3**) suggested directionally lower risks of suicidal ideation or attempts among female patients and those aged 45–64 years, whereas findings among individuals aged ≥65 years and those<45 years were neutral. These observations may be explained by limited statistical power due to very low event numbers, as well as competing mental health risk factors in older adults. Metabolic subgroups demonstrated similar patterns, with patients having HbA1c<7% or eGFR ≥60 mL/min/1.73 m² showing trends toward lower risk. These associations are biologically plausible given the links between metabolic health, quality of life, and psychological well-being; however, all subgroup findings should be regarded as exploratory.

Our findings align with previous observational studies on GLP-1 RAs, which generally report no significant association between these medications and increased suicide risk (12–15). Interestingly, Almeida et al. found that individuals exposed to GLP-1 RAs were at a greater risk of being prescribed antidepressants, suggesting a potential influence of GLP-1 RAs on mood. While this might seem contradictory to our findings, it is important to note that increased antidepressant use does not necessarily equate to increased suicidal ideation or attempts. In fact, antidepressant prescriptions may reflect proactive management of underlying depression, potentially reducing the risk of severe outcomes like suicide (21). Given the high prevalence of pre-existing depression and anxiety in our cohort, concomitant antidepressant or other psychotropic medication use is likely common. However, incomplete capture of medication adherence, dosage changes, and polypharmacy within TriNetX may introduce residual confounding.

The psychiatric safety observed in our study is consistent with findings from major RCTs involving tirzepatide, such as the SURMOUNT trials (7, 8, 22). These studies primarily focused on weight reduction and metabolic outcomes, but they also reported improvements in overall quality of life. While the primary outcomes of these trials did not specifically assess suicidal ideation or behaviors, the significant weight loss achieved with tirzepatide could have positively impacted patients’ psychological well-being, indirectly reducing the risk of mental health-related adverse outcomes. The substantial weight reduction observed in the SURMOUNT trials, up to 20%, is well-established to improve self-esteem, body image, and physical functioning, which are key factors in mitigating suicide risk. Our findings, combined with data from the SURMOUNT studies, suggest that tirzepatide’s comprehensive benefits on metabolic and psychological health make it a safe and effective option for patients with obesity, including those at risk for mental health issues.

The reduction in suicidal ideation and attempts observed in patients treated with tirzepatide could be explained by several biological mechanisms. GLP-1 receptor agonists, including tirzepatide, are thought to exert neuroprotective effects through the modulation of neuroinflammation, the hypothalamic-pituitary-adrenal (HPA) axis, and neurotransmitter pathways, which play key roles in mood regulation and stress response (23). Tirzepatide’s dual agonism, targeting both GLP-1 and GIP receptors, may provide additional benefits (24). While GLP-1 receptor agonists have shown potential in mood stabilization, the GIP pathway may further enhance emotional and cognitive outcomes, potentially contributing to the observed reduction in suicide risk (25). GIP signaling, though less studied in psychiatric contexts, has been linked to improved cognitive processes in animal models, and this dual pathway activation could offer unique neuropsychiatric benefits (26, 27).

The study has several strengths, including the use of a large, real-world database from the TriNetX network, which enhances the generalizability of our findings. We applied rigorous methodology, including PSM, and compared tirzepatide with other active anti-obesity medications to ensure balanced and robust comparisons. Moreover, the inclusion of a wide range of covariates, such as socioeconomic status and multiple comorbidities (e.g., depression, anxiety), enabled a comprehensive analysis. Finally, we evaluated suicide risk across multiple time frames, 3, 6, and 12 months, offering valuable insights into both the short- and intermediate-term effects of tirzepatide.

Nonetheless, our study has several limitations. First, as a retrospective observational analysis using EHR data, residual confounding is possible, even though we employed PSM and adjusted for known psychiatric and metabolic covariates, as well as socioeconomic status and lifestyle-related factors (smoking and alcohol use). However, we were unable to account for the severity of psychiatric illness or healthcare access, which may influence both treatment allocation and suicidality risk. Second, we excluded patients with prior suicidal ideation or suicide attempts to reduce confounding, but this prevented us from evaluating whether tirzepatide could reduce suicidality in that high-risk subset. Third, the low event rate, 17 cases in the tirzepatide group and 33 in the control group, limits statistical power and magnifies the impact of potential misclassification. In addition, reliance on diagnostic coding may underestimate the true incidence of suicidal ideation, as sensitivity and specificity of such codes are uncertain. Fourth, our follow-up period was only one year, which may not capture longer-term or delayed psychiatric events. In addition, we did not separately analyze censoring due to treatment discontinuation, switching, or death, and no competing-risks approach was applied. Given the low number of events and platform constraints, such analyses were not feasible. Fifth, while TriNetX spans multiple healthcare organizations, its data primarily derive from U.S. health systems and may not fully generalize to underserved or non-U.S. populations. Sixth, we did not perform imputation for missing data. Analyses were conducted using available values within the TriNetX platform, and patients with missing covariates were excluded from the relevant variable-specific analyses. This may introduce bias if missingness was not random. Seventh, consistent weight-loss data and patient-reported measures of mood or treatment satisfaction were not available in the TriNetX platform. This may limit our ability to evaluate whether the observed protective association is mediated by improvements in psychological well-being. Finally, our subgroup analyses did not use multiple-comparison corrections and should be regarded as exploratory, particularly given the small number of events. In addition, TriNetX’s privacy policy required rounding of event counts<10 to 10, which may slightly distort reported incidence rates but does not materially affect HR estimates derived from Cox models.

In conclusion, our results indicate that tirzepatide was not associated with an increased risk of suicidal ideation or suicide attempts. Although a HR below unity suggests a possible protective association, the absolute risk reduction was very small, and this finding should be considered tentative and hypothesis-generating. The key clinical message is that no safety signal for increased suicidality was observed. Longer-term prospective studies or randomized clinical trials with psychiatric endpoints are warranted to validate this association and to explore potential biological mechanisms in greater detail.

## Data Availability

This population-based study obtained data from the TrinetX platform (accessible at https://trinetx.com/), for which third-party restrictions apply to the availability of this data. The data were used under license for this study with restrictions that do not allow for data to be redistributed or made publicly available. To gain access to the data, a request can be made to TriNetX (join@trinetx.com), but costs might be incurred, and a data-sharing agreement would be necessary.

## References

[1] Koliaki C Dalamaga M Liatis S . Update on the obesity epidemic: after the sudden rise, is the upward trajectory beginning to flatten? Curr Obes Rep. (2023) 12:514–27. doi: 10.1007/s13679-023-00527-y, PMID: 37779155 PMC10748771

[2] Flegal KM Kit BK Orpana H Graubard BI . Association of all-cause mortality with overweight and obesity using standard body mass index categories: A systematic review and meta-analysis. JAMA. (2013) 309:71. doi: 10.1001/jama.2012.113905, PMID: 23280227 PMC4855514

[3] Apovian CM . Obesity: definition, comorbidities, causes, and burden. Am J Manag Care. (2016) 22:s176–185., PMID: 27356115

[4] Luppino FS De Wit LM Bouvy PF Stijnen T Cuijpers P Penninx BW . Overweight, obesity, and depression: A systematic review and meta-analysis of longitudinal studies. Arch Gen Psychiatry. (2010) 67:220. doi: 10.1001/archgenpsychiatry.2010.2, PMID: 20194822

[5] Graham C Frisco M . The relationship between obesity and suicide ideation among young adults in the United States. SSM - Popul Health. (2022) 18:101106. doi: 10.1016/j.ssmph.2022.101106, PMID: 35539364 PMC9079098

[6] Klinitzke G Steinig J Blüher M Kersting A Wagner B . Obesity and suicide risk in adults—A systematic review. J Affect Disord. (2013) 145:277–84. doi: 10.1016/j.jad.2012.07.010, PMID: 22871535

[7] Jastreboff AM Aronne LJ Ahmad NN Wharton S Connery L Alves B . Tirzepatide once weekly for the treatment of obesity. N Engl J Med. (2022) 387:205–16. doi: 10.1056/NEJMoa2206038, PMID: 35658024

[8] Zhao L Cheng Z Lu Y Liu M Chen H Zhang M . Tirzepatide for weight reduction in Chinese adults with obesity: the SURMOUNT-CN randomized clinical trial. JAMA. (2024) 332:551–60. doi: 10.1001/jama.2024.9217, PMID: 38819983 PMC11337071

[9] Pan X Tan B Chin YH Lee ECZ Kong G Chong B . Efficacy and safety of tirzepatide, GLP -1 receptor agonists, and other weight loss drugs in overweight and obesity: a network meta-analysis. Obesity. (2024) 32:840–56. doi: 10.1002/oby.24002, PMID: 38413012

[10] European Medicines Agency . EMA statement on ongoing review of GLP-1 receptor agonists(2023). Available online at: www.ema.europa.eu/en/news/ema-statement-ongoing-review-glp-1receptor-agonists (Accessed September 1, 2025).

[11] Tobaiqy M Elkout H . Psychiatric adverse events associated with semaglutide, liraglutide and tirzepatide: a pharmacovigilance analysis of individual case safety reports submitted to the EudraVigilance database. Int J Clin Pharm. (2024) 46:488–95. doi: 10.1007/s11096-023-01694-7, PMID: 38265519 PMC10960895

[12] Wang W Volkow ND Berger NA Davis PB Kaelber DC Xu R . Association of semaglutide with risk of suicidal ideation in a real-world cohort. Nat Med. (2024) 30:168–76. doi: 10.1038/s41591-023-02672-2, PMID: 38182782 PMC11034947

[13] Ueda P Söderling J Wintzell V Svanström H Pazzagli L Eliasson B . GLP-1 receptor agonist use and risk of suicide death. JAMA Intern Med. (2024) 184:e244369. doi: 10.1001/jamainternmed.2024.4369, PMID: 39226030 PMC11372654

[14] Tang H Lu Y Donahoo WT Shao H Shi L Fonseca VA . Glucagon-like peptide-1 receptor agonists and risk for suicidal ideation and behaviors in U.S. Older adults with type 2 diabetes: A target trial emulation study. Ann Intern Med. (2024) 177:1004–15. doi: 10.7326/M24-0329, PMID: 39008852 PMC12132802

[15] Hurtado I Robles C Peiró S García-Sempere A Sanfélix-Gimeno G . Association of glucagon-like peptide-1 receptor agonists with suicidal ideation and self-injury in individuals with diabetes and obesity: a propensity-weighted, population-based cohort study. Diabetologia. (2024) 67:2471–80. doi: 10.1007/s00125-024-06243-z, PMID: 39103719 PMC11519213

[16] Hung A Maciejewski ML Berkowitz TSZ Arterburn DE Mitchell JE Bradley KA . Bariatric surgery and suicide risk in patients with obesity. Ann Surg. (2023) 278:e760–5. doi: 10.1097/SLA.0000000000005825, PMID: 36805965 PMC10440362

[17] Cabanas-Sánchez V Yu T Rodríguez-Artalejo F Martínez-Gómez D . Weight loss as a risk factor for suicide. A prospective cohort study in more than 200,000 adults. Obes Res Clin Pract. (2023) 17:269–70. doi: 10.1016/j.orcp.2023.04.002, PMID: 37059616

[18] Taquet M Dercon Q Todd JA Harrison PJ . The recombinant shingles vaccine is associated with lower risk of dementia. Nat Med. (2024) 30:2777–81. doi: 10.1038/s41591-024-03201-5, PMID: 39053634 PMC11485228

[19] Huang LA Lo SC Yang YS Huang CN Wang CC Wang YH . Association of COVID-19 infection with subsequent thyroid dysfunction: an international population-based propensity score matched analysis. Thyroid Off J Am Thyroid Assoc. (2024) 34:442–9. doi: 10.1089/thy.2023.0626, PMID: 38407979

[20] TrinetX . Trinetx-publication-guidelines. Available online at: https://trinetx.com/real-world-resources/case-studies-publications/trinetx-publication-guidelines/ (Accessed September 1, 2025).

[21] Almeida OP Fong Z Hill Almeida LM Sanfilippo FM Page A Etherton-Beer C . Cross-sectional, case-control and longitudinal associations between exposure to glucagon-like peptide-1 receptor agonists and the dispensing of antidepressants. Diabetes Obes Metab. (2024) 26:2925–32. doi: 10.1111/dom.15616, PMID: 38650544

[22] Malhotra A Grunstein RR Fietze I Weaver TE Redline S Azarbarzin A . Tirzepatide for the treatment of obstructive sleep apnea and obesity. N Engl J Med. (2024) 391:1193–205. doi: 10.1056/NEJMoa2404881, PMID: 38912654 PMC11598664

[23] Hölscher C . Central effects of GLP-1: new opportunities for treatments of neurodegenerative diseases. J Endocrinol. (2014) 221:T31–41. doi: 10.1530/JOE-13-0221, PMID: 23999914

[24] Seino Y Fukushima M Yabe D . GIP and GLP-1, the two incretin hormones: Similarities and differences. J Diabetes Investig. (2010) 1:8–23. doi: 10.1111/j.2040-1124.2010.00022.x, PMID: 24843404 PMC4020673

[25] Zheng Z Zong Y Ma Y Tian Y Pang Y Zhang C . Glucagon-like peptide-1 receptor: mechanisms and advances in therapy. Signal Transduct Target Ther. (2024) 9:234. doi: 10.1038/s41392-024-01931-z, PMID: 39289339 PMC11408715

[26] Nyberg J Jacobsson C Anderson MF Eriksson PS . Immunohistochemical distribution of glucose-dependent insulinotropic polypeptide in the adult rat brain. J Neurosci Res. (2007) 85:2099–119. doi: 10.1002/jnr.21349, PMID: 17510976

[27] Ding KH Zhong Q Xie D Chen HX Della-Fera MA Bollag RJ . Effects of glucose-dependent insulinotropic peptide on behavior. Peptides. (2006) 27:2750–5. doi: 10.1016/j.peptides.2006.05.011, PMID: 16822587

