# Supplementary material for: Association of tirzepatide and the risk of suicide in a real-world cohort
Source: Front Psychiatry. 2025 Nov 17;16:1626103. doi: 10.3389/fpsyt.2025.1626103 (PMC12667738; doi:10.3389/fpsyt.2025.1626103)
Supplement: Supplementary file 1 [file Table1.docx]

| **Supplemental table 1**. Detailed coding of this study | |
| --- | --- |
| **Outcomes** |  |
| Suicidal ideations | Suicidal ideations (ICD-10 code: R45.851) |
| Suicide attempt | Suicide attempt (ICD-10 code: T14.91) |
|  |  |
| **Study population** |  |
| Overweight or obesity | Overweight and obesity (ICD-10 code: E66) Body mass index [BMI] 40 or greater, adult) ICD-10 code: Z68.4 Body mass index [BMI] 30-39, adult) ICD-10 code: Z68.3 Body mass index [BMI] 25-25.9, adult) ICD-10 code: Z68.25 Body mass index [BMI] 26-26.9, adult) ICD-10 code: Z68.26 Body mass index [BMI] 27-27.9, adult) ICD-10 code: Z68.27 Body mass index [BMI] 28-28.9, adult) ICD-10 code: Z68.28 Body mass index [BMI] 29-29.9, adult) ICD-10 code: Z68.29 |
|  |  |
| **Cases** |  |
| Tirzepatide | Rxnorm code: 2601723 |
|  |  |
| **Controls** |  |
| Non-GLP1R agonists anti-obesity  medication | Orlistat (RxNorm code:37925), Phentermine (RxNorm code:8152), Topiramate (RxNorm code:38404), Bupropion (RxNorm code:42347), Naltrexone (RxNorm code:7243) |
|  |  |
| **Other coding and comorbidities** |  |
| T2DM | Type 2 diabetes mellitus )ICD-10 code: E11) |
| Other GLP1R agonists medications | Lixisenatide (RxNorm code: 1440051), Albiglutide (RxNorm code: 1534763), Dulaglutide (RxNorm code: 1551291), Liraglutide (RxNorm code: 475968), Exenatide (RxNorm code: 60548), Semaglutide (RxNorm code: 1991302) |
| Non-GLP1R agonists anti-diabetes medications | Drugs used in diabetes (ATC code: A10) with GLP1R agonists excluded |
| Medications related to suicidal ideation pharmacotherapy | Antidepressants (ATC code: N06A), Antipsychotics (ATC code: N05A), Antiepileptics (ATC code: N03), Benzodiazepine derivative sedative/hypnotics (VA code: CN302), Esketamine (RxNorm code: 2119365), Ketamine (RxNorm code: 6130), Lithium (RxNorm code: 6448), |
| Age at the index event | Age |
| Female | F |
| Male | M |
| Asian | Asian (Demographics: 2028-9) |
| Black or African American | Black or African American (Demographics: 2054-5) |
| White | White (Demographics: 2106-3) |
| Hispanic/latino | Hispanic/latino (Demographics: 2135-2) |
| Not Hispanic/latino | Not Hispanic/latino (Demographics: 2186-5) |
| Unknown race | Unknown race (Demographics: 2131-1) |
| Unknown ethnicity | Unknown ethnicity (Demographics: UN) |
| Divorced | Divorced (Demographics: D) |
| Widowed | Widowed (Demographics: W) |
| Never married | Never married (Demographics: S) |
| Adverse socioeconomic and psychosocial circumstances | Persons with potential health hazard related to socioeconomic and psychosocial circumstances (ICD-10 code: Z55-Z65), including problems related to education (Z55), employment/unemployement (Z56), housing and economic circumstances (Z59), social environment (Z60), upbringing (Z62), family circumstances (Z63), psychosocial circumstances (Z64, Z65) |
| Personal history of psychological trauma | Personal history of psychological trauma, not elsewhere classified (ICD-10 code: Z91.4) |
| Family history of mental disorders | Family history of mental behaviour disorders (ICD-10: Z81) |
| Problems related to lifestyle | Problems related to lifestyle (ICD-10 code: Z72) |
| Depression | Depressive episode (ICD-10 code: F32) |
| Mood disorders | Mood [affective] disorders (ICD-10 code: F30-F39) |
| Anxiety | Anxiety, dissociative, stress-related, somatoform and other nonpsychotic mental disorder (ICD-10 : F40-F48) |
| Psychotic disorders | Schizophrenia, schizotypal, delusional, and other non-mood psychotic disorders (ICD-10 : F20-F29) |
| Behavioral disorders | Behavioural syndromes associated with physiolgical disturbances and physical factors (ICD-10 code: F50-F59) |
| Disorders of adult personality and behavior | Disorders of adult personality and behavior (ICD-10: F60-F69) |
| Sleeping disorders including insomnia | Sleeping disorders (ICD-10 code: G47) |
| Intentional self-harm | Intentional self-harm (ICD-10 code: X71-X83) |
| Personal history of self-harm | Personal history of self-harm (ICD-10 code: Z91.5) |
| Chronic pain | Chronic pain, not elsewhere classified (ICD-10 code:G89.2) |
| Alcohol use disorder | Alcohol use disorder (ICD-10 : F10) |
| Tobacco use disorder | Nicotine dependence (ICD-10 code: F17) |
| Opioid use disorder | Opioid use disorders (ICD-10 code: F11) |
| Cannabis use disorder | Cannabis use disorders (ICD-10 code: F12) |
| Cocaine use disorder | Cocaine use disorder (ICD-10 code: F14) |
| Other stimulant disorder | Other stimulant disorder (ICD-10 code: F15) |
| Other psychoactive substance use disorders | Other psychoactive substance related disorders (ICD-10 code: F19) |
| Cancer | Neoplasms (ICD-10 code: C00-D49) |
| Traumatic brain injury | Intracranial injury (ICD-10 code: S06) |
| Bariatric surgery | Bariatric surgery (ICD-10 code: Z98.84) |
| Insulins | Insulins and analogues (ATC code: A10A) |
| Meformin | Metformin (RxNorm code:6809) |
| Sulfonylureas | Sulfonylureas (ATC code: A10BB) |
| Alpha glucosidase inhibitors | Alpha glucosidase inhibitors (ATC code: A10BF) |
| Thiazolidinedione | Thiazolidinedione (ATC code: A10BG) |
| Dipeptidyl peptidase 4 (DPP-4) inhibitors | Dipeptidyl peptidase 4 (DPP-4) inhibitors (ATC code: A10BH) |
| SGLT2 inhibitors | Sodium-glucose co-transporter 2 (SGLT2) inhibitors (ATC code: A10BK) |
